# Oral appliance therapy versus nasal continuous positive airway pressure in obstructive sleep apnea: A randomized, placebo‐controlled trial on temporomandibular side‐effects

**DOI:** 10.1002/cre2.288

**Published:** 2020-04-04

**Authors:** Maria Nikolopoulou, Ghizlane Aarab, Jari Ahlberg, Hans L. Hamburger, Jan de Lange, Frank Lobbezoo

**Affiliations:** ^1^ Department of Orofacial Pain and Dysfunction, Academic Centre of Dentistry Amsterdam (ACTA) University of Amsterdam and Vrije Universiteit Amsterdam Amsterdam The Netherlands; ^2^ Department of Oral and Maxillofacial Diseases University of Helsinki Helsinki Finland; ^3^ Amsterdam Sleep Centre, Boerhaave Medical Centre Amsterdam The Netherlands; ^4^ Department of Oral and Maxillofacial Surgery Amsterdam University Medical Centre and Academic Centre for Dentistry Amsterdam (ACTA) Amsterdam The Netherlands

**Keywords:** continuous positive airway pressure, oral appliance, side‐effects, sleep apnea, temporomandibular disorders

## Abstract

**Purpose:**

To assess the differences in the frequency of clinical signs of temporomandibular disorder (TMD) pain and mandibular function impairment between mandibular advancement device (MAD) and nasal continuous positive airway pressure (nCPAP) therapies in obstructive sleep apnea (OSA) patients at baseline and after 6 month of treatment.

**Methods:**

This study concerns a secondary analysis of a randomized placebo‐controlled trial in which different treatment effects of an objectively titrated MAD were compared with those of nCPAP and an intra‐oral placebo appliance in a parallel design. Sixty‐four mild to severe OSA patients (52.0 ± 9.6 years) were randomly assigned to these three groups. All patients underwent a shortened functional examination of their masticatory system at baseline and after 6 months to establish the presence of clinical signs of TMD pain. Mandibular function impairment was assessed with a questionnaire.

**Results:**

Clinical signs of TMD pain were only rarely present at baseline and therapy evaluation. No significant differences were found between the three groups in the (low) frequency of clinical signs of TMD pain at both time points (*p* = .401–.176). In addition, the (low) scores of mandibular function impairment did not differ between the three groups either, neither at baseline (*p* = .744) nor after 6 months (*p* = .359).

**Conclusions:**

A low frequency of clinical signs of TMD pain in mild to severe OSA patients was found after 6 months, regardless of treatment with MAD or nCPAP. In addition, no difference in mandibular function impairment was observed between the different treatment modalities.

## 1 INTRODUCTION

Obstructive sleep apnea (OSA) is characterized by recurrent obstructions of the upper airway, often resulting in oxygen desaturations and arousals from sleep (American Academy of Sleep Medicine, 1999). OSA is a common sleep‐related breathing disorder that affects 10–17% of middle‐aged men and 3–9% of middle‐aged women, with a higher prevalence amongst obese patients (Badran, Ayas, & Laher, 2014). OSA patients without effective treatment have an increased risk of cardiovascular conditions like hypertension, stroke, heart failure, and atrial fibrillation (Ayas, Owens, & Kheirandish‐Gozal, 2015; Marin, Carrizo, Vicente, & Agusti, 2005; Yaggi et al., 2005).

The treatment of OSA has been undergoing a steady shift over the last years. While (nasal) CPAP ([n]CPAP) was more or less the sole effective treatment for many years, mandibular advancement device (MAD) therapy is increasingly recognized as a viable treatment for OSA (Aarab, Lobbezoo, Hamburger, & Naeije, 2011; Ramar et al., 2015). MADs are currently indicated for the treatment of mild to moderate OSA patients as well as of severe OSA patients who are intolerant to or refuse CPAP therapy (Aarab, Lobbezoo, Hamburger, & Naeije, 2011; Ramar et al., 2015). MADs protrude the mandible and improve upper airway patency by enlarging the upper airway and/or by reducing its collapsibility (Schmidt‐Nowara et al., 1995). During the monitoring phase of this treatment, the mandibular protrusion position of the MAD is often titrated by the dentist or patient to improve its efficacy and to reduce its side‐effects (Aarab, Lobbezoo, Hamburger, & Naeije, 2010). However, due to their design, MADs exert potentially detrimental forces on the teeth, oral soft tissues, and musculoskeletal structures of the masticatory system. Amongst others, MADs may result in excessive salivation, mouth dryness, and temporomandibular side‐effects in the short‐term (Doff et al., 2012; Hammond et al., 2007; Martinez‐Gomis et al., 2010; Pantin, Hillman, & Tennant, 1999; Tegelberg et al., 1999).

Temporomandibular disorders (TMDs) are defined as musculoskeletal disorders that include symptoms like pain and dysfunction of the temporomandibular joint and/or the jaw muscles (de Leeuw & Klasser, 2018). De Leeuw and Klasser (2018) extensively describe various methods for the clinical assessment of TMD pain, all of them based on a combination of self‐report and clinical tests that provoke the musculoskeletal system. Importantly, the clinical assessment of the impairment of mandibular function associated with TMDs should not only comprise a diagnostic assessment of symptoms and signs but also an assessment of the functional impairment as it is perceived in the patient's value system (Stegenga, de Bont, de Leeuw, & Boering, 1993).

Both improvements and deteriorations in signs and symptoms of TMDs have been found during MAD treatment (Bondemark & Lindman, 2000; Cunali et al., 2009; Doff et al., 2012; Fransson, Tegelberg, Leissner, Wenneberg, & Isacsson, 2003; Giannasi et al., 2009; Martinez‐Gomis et al., 2010; Petit et al., 2002). Most previous studies, however, were retrospective in design or did not include a placebo group (Bondemark & Lindman, 2000; Cunali et al., 2009; Doff et al., 2012; Martinez‐Gomis et al., 2010). Moreover, the impact of the TMD on the patient's mandibular function has seldom been determined (Doff et al., 2012). Therefore, a definitive conclusion about the frequency of TMD side‐effects and their impact on mandibular function in OSA patients under MAD treatment cannot be drawn.

The aim of this study was to assess the differences in the frequency of clinical signs of TMD pain and in the mandibular function impairment between MAD and nCPAP therapies at baseline and after 6 months in mild to severe OSA patients in a randomized, placebo‐controlled trial design. We hypothesized that an MAD would result in significantly more clinical signs of TMD pain than nCPAP and placebo. Further, this TMD pain was hypothesized to lead to more mandibular function impairment in the MAD group than in the nCPAP and placebo groups.

## 2 MATERIALS AND METHODS

This study concerns a secondary analysis of a large randomized placebo‐controlled trial in which different treatment effects of an objectively titrated MAD were compared with those of nCPAP and an intra‐oral placebo appliance in a parallel design. The short‐term and long‐term outcomes of this trial have been published previously (Aarab et al., 2010; Aarab, Lobbezoo, Hamburger, & Naeije, 2011; Aarab, Lobbezoo, Heijmans, Hamburger, & Naeije, 2011).

### 2.1 Participants

**FIGURE 1:**
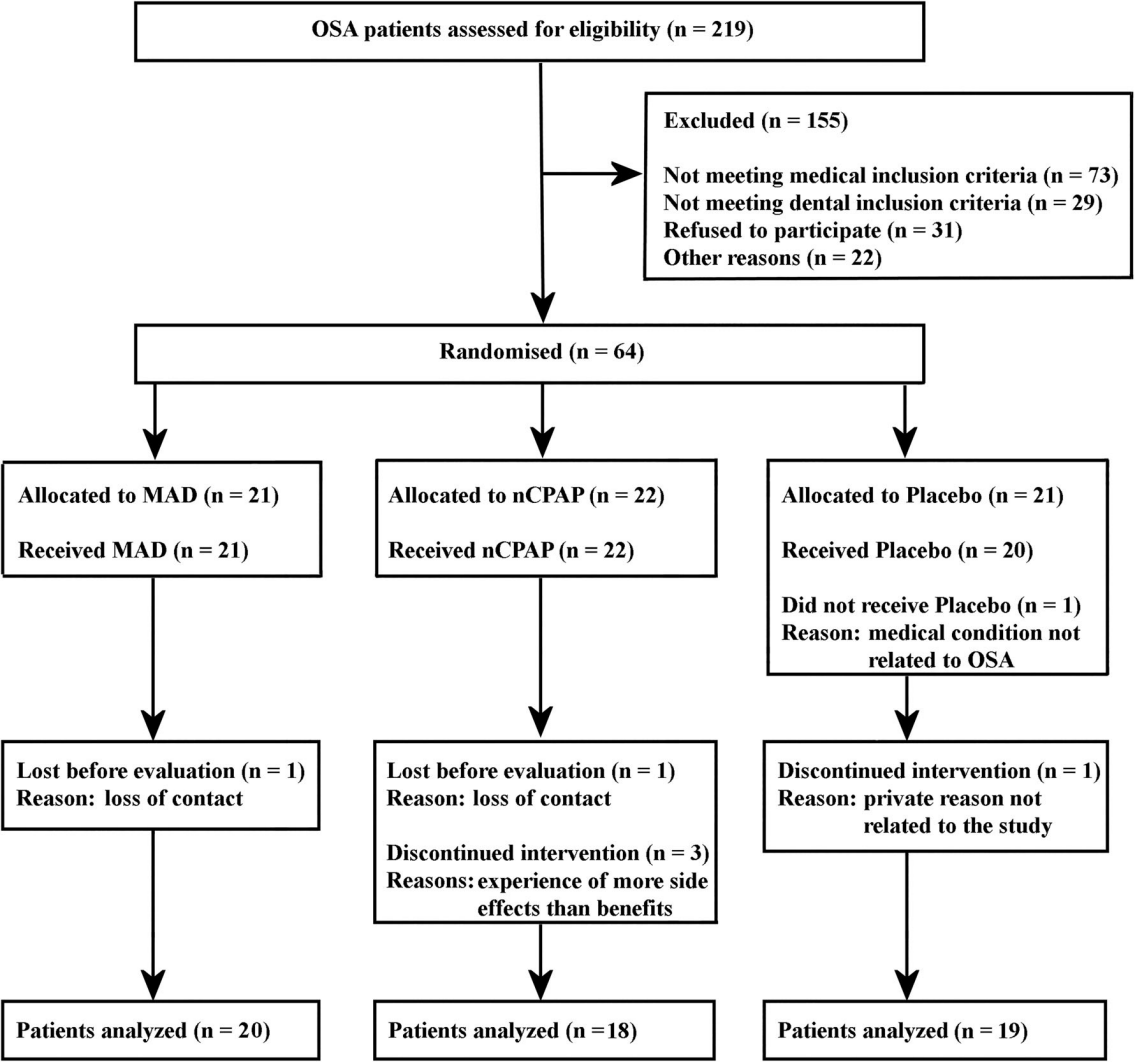
Flow‐chart of the patients through each stage of the trial. MAD, mandibular advancement device; nCPAP, nasal continuous positive airway pressure; OSA, obstructive sleep apnea

Potential participants were recruited from the Center for Sleep–Wake Disorders of the Slotervaart Medical Center in Amsterdam, The Netherlands. The multidisciplinary team of this center consisted of a neurologist, ENT specialists, pulmonologists, a dentist, psychologists, and sleep medicine technicians. All participants were at least 18 years old, with an apnea‐hypopnea index (AHI) of 5–45 events/hr. They all reported excessive daytime sleepiness (Epworth sleepiness score ≥ 10), or at least two OSA symptoms presented by the American Academy of Sleep Medicine Task Force (e.g., daytime sleepiness, fatigue) (American Academy of Sleep Medicine, 1999). The exclusion criteria were the existence of sleep disorders other than OSA based on polysomnography, a body mass index (BMI) of more than 40, usage of medication that affects sleep or respiration, reversible morphological upper airway abnormalities, and previous treatment with nCPAP or an intraoral appliance. Patients with clinical signs of temporomandibular disorders (TMDs; diagnosis based on a functional examination of the masticatory system; Visscher et al., 2009) who also expressed a desire for treatment of their TMD complaints, an unhealthy periodontium (periodontal pockets >5 mm), dental pain, and/or an inadequate retention possibilities for an intra‐oral appliance were excluded as well. Two‐hundred‐nineteen participants were eligible for the study. Seventy‐three of them did not meet the medical inclusion criteria, and 29 patients did not meet the dental inclusion criteria. Thirty‐one patients refused to participate and 22 patients did not participate for other reasons, for example, loss of contact. Finally, a total of 64 OSA patients agreed to participate and provided written informed consent (Figure 1). The scientific and ethical aspects of the protocol were reviewed and approved by the Medical Ethics Committee of the Slotervaart Medical Center (## U/1731/0326, U/2679/0326). This study has been registered at www.clinicaltrials.gov (# NCT00950495).

### 2.2 Interventions

The nCPAP group used the REMstar Pro system (Respironics, Herrsching, Germany). The MAD group used a custom‐made device with an individually adjustable mandibular protrusion position at a constant vertical dimension, the design of which has been described in detail previously (Aarab et al., 2010). The MAD did not allow vertical opening and lateral movements. The placebo group used a thin (˂1 mm) hard acrylic resin palatal splint with only a partial coverage of the hard palate and no interference with the dental occlusion (Aarab, Lobbezoo, Hamburger, & Naeije, 2011).

### 2.3 Study protocol

The protocol of this study has been described in detail previously (Aarab, Lobbezoo, Hamburger, & Naeije, 2011). Below, an outline is provided with the protocol's key characteristics, along with pertinent additions that made it possible to answer the current research question.

All patients were randomly allocated to one of the three therapy groups. To ensure that the groups were of approximately the same size, block randomization was used. Block sizes were 6, 12, and 18; sizes were randomly varied. The allocation sequence was automatically generated and subsequently concealed by an independent co‐worker, who kept a paper copy in a lockable drawer. Sealed opaque envelopes were used to conceal the allocation from the principal investigator (Aarab, Lobbezoo, Hamburger, & Naeije, 2011). Both MAD and nCPAP were titrated before the start of the treatment (Aarab, Lobbezoo, Hamburger, & Naeije, 2011). For the titration of the MAD, four ambulatory polysomnographic (PSG) recordings were performed at regular time intervals of approximately 3 weeks. The total titration period was approximately 10 weeks. The most effective protrusion position of the MAD (i.e., the mandibular position that yielded the lowest AHI value) was chosen from among four randomly offered positions, namely, 0, 25, 50, and 75% of the maximum protrusion. The MAD was set at 25% of the maximum protrusion in one patient, at 50% in 7 patients, and 75% in 12 patients (Aarab, Lobbezoo, Hamburger, & Naeije, 2011). The titration of nCPAP was performed during a PSG recording at the Slotervaart Medical Center. The pressure was increased in steps of 1 cm H_2_O/hr, until the AHI and respiration‐related arousals were reduced to ≤5 events/hr, and snoring was minimized. The average value of the pressure was 7.3 (SD, 1.9; range, 4–11) cm H_2_O (Aarab, Lobbezoo, Hamburger, & Naeije, 2011). For the placebo group, four ambulatory PSG recordings were performed at regular time intervals similar to the MAD group (Aarab, Lobbezoo, Hamburger, & Naeije, 2011).

During the titration period of approximately 10 weeks, all patients visited ACTA four times at regular intervals, during which the BMI (kg/m^2^) was determined and the Epworth Sleepiness Scale (ESS) was completed (Johns, 1991). The participants were also interviewed about their compliance (% of nights per week of wearing), and the change (increased, unchanged, or decreased) in snoring intensity, based on information they obtained from their bed partner. These outcomes have been described in detail by Aarab, Lobbezoo, Hamburger, and Naeije (2011). Further, the visits at ACTA were also used to adjust the protrusion position of the MAD according to the random order of the study protocol.

Besides the above‐described titration PSGs, all three groups underwent two full PSG recordings in the sleep laboratory of the Slotervaart Medical Center: The first one before treatment and the second one 6.0 ± 2.0 months (mean ± SD) after the start of the treatment. The outcomes of the PSG recordings have been published by Aarab et al. (2011).

### 2.4 Clinical signs of TMD pain and mandibular function impairment

During the consultations at baseline and after 6 months of treatment, patients were informed about the mild and transient nature of a possible TMD pain by the clinician. The assessment of TMD pain and mandibular function impairment was performed at both time points. The assessment included, amongst others, an oral history and orthopedic tests, namely, the static and dynamic tests (Visscher et al., 2009). A single, experienced, and well‐trained clinician performed all examinations throughout the entire study. This clinician was not blinded for the type of treatment of each patient. Clinical signs of TMD pain was considered present when patients reported pain on at least one of the static or dynamic tests during opening, closing, and protrusion of the mandible. The presence of clinical signs of TMD pain was scored “1”, and their absence was scored “0”.

The Mandibular Function Impairment Questionnaire (MFIQ) was completed by all patients at baseline and after 6 months, to subjectively assess function impairment of the masticatory system. The MFIQ is a validated questionnaire, which is used to assess the impact of TMDs on mandibular function in daily life (Stegenga et al., 1993). The MFIQ scores perceived difficulty of 17 representative mandibular functions in relation to joint or muscle complaints. The answers are scored on five‐point Likert‐type scales (0–4), where 0 represents “no difficulty” and 4 represents “very great difficulty or impossible without help”. The sum item score for function impairment ranges from 0 to 68. Using these scores, a Raw Component Score is calculated and a functional impairment rating scale (FIRS) is derived (0–5). A FIRS of 0 or 1 indicates low level of function impairment, a FIRS of 2 or 3 indicates moderate level of function impairment, and a FIRS of 4 or 5 indicates severe level of function impairment.

### 2.5 Statistical analysis

The null hypothesis of this study was that there is no significant difference between the MAD group, the nCPAP group, and the placebo group in the presence of clinical signs of TMD pain at baseline and at therapy evaluation. The chi‐square (*X* ^2^) test was used to examine whether the distributions of TMD pain between the three groups differed. The Wilcoxon Signed rank test (for the within groups comparison) and the Kruskal–Wallis tests (for the between‐groups comparison) were used to test the difference between the three groups in the change of their FIRS score between baseline and 6 months after the start of the treatment. All statistical analyses were performed using the Statistical Package for the Social Sciences (version 24.0, SPSS Inc., Chicago, IL). *p* < .05 was considered statistically significant.

## 3 RESULTS

The patient characteristics at baseline are presented in Table 1. BMI was the only baseline characteristic that differed between the three therapy groups. Seven patients dropped out of the study for various reasons (Figure 1). Thus, 57 participants (20 MAD patients, 18 nCPAP patients, and 19 placebo patients) completed the entire study protocol (Aarab, Lobbezoo, Hamburger, & Naeije, 2011).

**TABLE 1:** Patient characteristics (mean ± SD) at baseline of the mandibular advancement device (MAD) group, the nasal continuous positive airway pressure (nCPAP) group, the placebo group, and the dropouts; see (Aarab, Lobbezoo, Hamburger, & Naeije, 2011) for more details

	MAD (*N* = 21)	nCPAP (*N* = 22)	Placebo (*N* = 21)	Dropouts (*N* = 7)
Age (years)	50.4 ± 8.9	54.0 ± 10.1	51.3 ± 9.6	49.3 ± 7.3
Number of men/women	17/4	15/7	15/6	5/2
Apnea‐hypopnea index (AHI)	21.4 ± 11.0	20.1 ± 9.0	19.5 ± 8.4	14.8 ± 3.8
Epworth sleepiness scale	12.0 ± 5.7	10.7 ± 4.4	10.8 ± 4.0	13.7 ± 1.9
Body mass Index^a^ (kg/m^2^)	27.1 ± 3.2	30.7 ± 3.7	31.1 ± 4.7	27.8 ± 4.1

a MAD patients had a significantly lower BMI than placebo and nCPAP patients (*p* = .002 and .006, respectively; one‐way ANOVA, followed by least‐significant difference pairwise comparisons).

Details of the primary analyses of the RCT have been described previously; see (Aarab, Lobbezoo, Hamburger, & Naeije, 2011). In short, the MAD group had used their appliance 90.6% (SD, 13.3) of the nights; the nCPAP group 82.9% (SD, 27.2) of the nights; and the placebo group 93.9% (SD, 15.7) of the nights. No significant group differences in compliance were found (*F* = 1.518, *p* = .228). In addition, BMI did not change significantly from baseline to 6‐month follow‐up in any of the three therapy groups (paired *t* tests; *p* = .408–.752). AHI, on the other hand, showed a significant improvement over time in all three therapy groups. The decrease in AHI from baseline to 6‐month follow‐up differed significantly between the groups (ANCOVA; *F* = 14.886, *p* = .000). While this decrease was comparable for MAD and nCPAP (*p* = .092), both treatments showed a significantly larger decrease than the placebo condition (*p* = .000 and .0002, respectively). Finally, for excessive daytime sleepiness, the pooled data of the three groups showed a significant decrease over time (paired *t* test, *p* = .002).

In Table 2, the outcome variables are presented. Clinical signs of TMD pain were only rarely encountered. No significant differences were found between the three treatment groups in the (low) frequency of the clinical signs of TMD pain at baseline and at therapy evaluation after 6 months (χ^2^ = 1.830 and χ^2^ = 3.478; *p* = .401 and .176, respectively). All FIRS scores were qualified as low. There was no significant change in the FIRS score within the groups between baseline and therapy evaluation (*Z* = −0.632; *p* = .527), nor was there a significant difference between the three different treatment groups in their (low) level of mandibular function impairment at baseline (*p* = .744) and after 6 months (*p* = .359; Table 2).

**TABLE 2:** Presence of clinical signs of TMD pain and the Function Impairment Rating Scale (FIRS) score at baseline and 6 months after the start of the therapy for the mandibular advancement device (MAD) group, the nasal continuous positive airway pressure (nCPAP) group, and the placebo group

Outcome measures	MAD (*n* = 20)		nCPAP (*n* = 18)		Placebo (*n* = 19)	
	Baseline	6 months	Baseline	6 months	Baseline	6 months
Presence of clinical signs of TMD pain (*n*)^a^	0	0	2	2	1	0
FIRS score (25%|median|75%)^b^	0|0|1	0|0|0.50	0|0|0.25	0|0|1	0|0|0	0|0|0

a Number of complete data sets per group: MAD (*n* = 18); nCPAP (*n* = 17); placebo (*n* = 14).

b Number of complete data sets per group: MAD (*n* = 17); nCPAP (*n* = 13); placebo (*n* = 18).

## 4 DISCUSSION

The aim of this study was to assess the differences in the frequency of clinical signs of TMD pain and mandibular function impairment after 6 months of treatment between MAD and nCPAP therapies in mild to severe OSA patients in a randomized, placebo‐controlled trial design. No significant differences were found between the three treatment groups in the frequency of clinical signs of TMD pain at baseline and at therapy evaluation after 6 months. Further, there was no significant difference between the three different treatment groups in their (low) level of mandibular function impairment in daily life either.

A study of Sanders et al. (2013) tested the hypothesis that signs and symptoms of OSA are associated with the occurrence of TMD, and precede first‐onset TMD. Their data was based on a prospective study (*n* = 2,604) and a case–control study (*n* = 1,716). Both studies supported a significant association between OSA symptoms and TMD, and they found evidence that OSA symptoms preceded first‐onset TMD. One of their explanations for OSA preceding TMD was that OSA patient shows more sleep bruxism (SB) activity and therefore more TMD. However, there is no solid evidence for the cause–effect relationship between OSA and SB on one hand, and between SB and TMD on the other hand (Manfredini, Guarda‐Nardini, Marchese‐Ragona, & Lobbezoo, 2015; Manfredini & Lobbezoo, 2010). Furthermore, OSA was not determined objectively (i.e., by means of PSG) in the study of Sanders et al. (2013). Therefore, their hypothesis should be tested further in future studies. Nevertheless, Kato et al. (2013) found self‐reported jaw symptoms (viz., morning jaw discomfort, morning jaw pain, daytime jaw pain, and jaw opening difficulties) in 19% of 511 OSA patients. Further, Perez et al. (2013) showed that TMD pain was present in approximately 10% of their OSA patients at baseline, based on a clinical examination, which corresponds with the TMD‐pain prevalence rate of 10% in the general population (LeResche, 1997). Based on these studies, we may conclude that TMD is associated with OSA. However, TMD pain may be equally prevalent in OSA patients and the general population.

The present study concerns a secondary analysis of a large randomized placebo‐controlled trial, the short‐term and long‐term outcomes of which have been published previously (Aarab et al., 2010; Aarab, Lobbezoo, Hamburger, & Naeije, 2011; Aarab, Lobbezoo, Heijmans, et al., 2011). This means that the data that were analyzed to answer the present research questions were originally collected for other purposes. While the advantages of using secondary data are clear (e.g., time‐saving, cost‐efficient), its use is also associated with potential disadvantages, such as the application outdated or inaccurate methods that may jeopardize the validity of the results. In the present study, however, both TMD pain and mandibular function impairment were assessed with up‐to‐date and validated tools, namely, static and dynamic tests (Visscher et al., 2009) and Mandibular Function Impairment Questionnaire (Stegenga et al., 1993), respectively. Hence, we are confident that in the present study, the use of secondary data has yielded accurate outcomes.

The experienced and well‐trained clinician who performed all examinations throughout the entire study was not blinded for the type of treatment of each patient. Since this approach is associated with a risk of observer bias, this could be considered as a potential limitation of the present study. Further, not all patients completed the entire protocol of the present study. Therefore, both our original study sample and the dropouts contributed to a reduced power of this study. Missing values may lead to selection bias, because participants who complete the entire study may show better treatment outcomes than dropouts (Donders, van der Heijden, Stijnen, & Moons, 2006). However, the outcomes of our study are similar to previous ones. Perez et al. (2013)determined the prevalence and incidence of TMD pain in 167 OSA patients undergoing MAD treatment. They found that after approx. 4 months, TMD pain was present in only a small proportion of their study sample and that this pain was no longer present after 1 year. Similar findings were reported by Doff et al. in their study wherein 51 MAD patients were compared to 52 CPAP patients on the occurrence of TMD and the risk of pain and function impairment in a 2‐year follow‐up (Doff et al., 2013). They found that MAD therapy is associated with increased TMD pain in the first 2 months of use, but that this TMD side‐effect had a transient nature: They found no difference in TMD pain between the MAD group and their CPAP group after 1 year. Therefore, they concluded from their study that, because of the transient nature of TMD pain, this pain is not a reason to contra‐indicate an MAD treatment. Also, Knappe, Bakke, Svanholt, Petersson, and Sonnesen (2017)) reported a low prevalence rate of jaw‐muscle tenderness, namely, 7.1%, and no significant changes in orofacial function in association with MAD therapy after 6 months. In our study, TMD pain in the MAD group was also evaluated after 6 months. We hypothesize, based on the outcomes of the studies of Perez et al. (2013), Doff et al. (2013), and Knappe et al. (2017), that the TMD pain in our MAD group had already disappeared in the first few months. Therefore, no difference in clinical signs of TMD pain between the MAD, nCPAP, and placebo groups was found in our study.

In conclusion, our study showed a low frequency of clinical signs of TMD pain in mild to severe OSA patients after 6 months, regardless of treatment with MAD or nCPAP. In addition, no difference in mandibular function impairment was observed between the different treatment modalities.

## CONFLICT OF INTEREST

Maria Nikolopoulou, Ghizlane Aarab, Jari Ahlberg, Hans Hamburger, and Jan de Lange declare that they have no conflict of interest. Frank Lobbezoo has received research grants from Sunstar Suisse S.A., SomnoMed‐Goedegebuure, and Airway Management. Frank Lobbezoo is member of the Academic Advisory Boards for GrindCare and Oral Function of Sunstar Suisse S.A.

## ETHICAL APPROVAL

All procedures performed in studies involving human participants were in accordance with the ethical standards of the institutional and/or national research committee and with the 1964 Helsinki declaration and its later amendments or comparable ethical standards.

## INFORMED CONSENT

Informed consent was obtained from all individual participants included in the study.
